# Comparison of the effects of high tibial osteotomy with and without a tourniquet

**DOI:** 10.1186/s12893-024-02681-z

**Published:** 2024-12-19

**Authors:** Huiwen Wu, Fangyuan Wang, Shihao Deng, Shuai Liang, Shaoze Lan, Kenan Sun, Ciren Lunzhu, Dawa Cangjue, Jun Li

**Affiliations:** 1 Present Address: Department of Orthopaedics, The Second Affiliated Hospital of Anhui Medical University, Hefei, 230601 China; 2 Institute of Orthopaedics, Research Center for Translational Medicine, The Second Affiliated Hospital of Anhui Medical University, Hefei, 230601 China; 3 Department of Orthopedics, Ningde City Mindong Hospital, Fu’an, 355000 China; 4 Present Address: Department of Orthopedics, Shannan City People’s Hospital, Shannan, 856004 China

**Keywords:** Knee, High tibial osteotomy, Tourniquet, Prognosis, Blood loss

## Abstract

**Background:**

Tourniquets are routinely employed to achieve hemostasis in modern limb surgeries. Nevertheless, the precise role and benefits of tourniquets in high tibial osteotomy (HTO) surgeries remain understudied. The aim of this study was to assess the application of tourniquets in high-tibial osteotomy procedures.

**Methods:**

This was a prospective study of patients who underwent HTO surgery at an identical hospital. The participants were randomly assigned into two groups: Group A, with a tourniquet (*n* = 43); and Group B, without a tourniquet (*n* = 43). The same surgical technique and postoperative care were employed for both groups of patients. Knee range of motion (ROM) and pain were assessed by utilizing a visual analogue scale (VAS) after exercise and maximum calf circumference, and postoperative Hospital for Special Surgery (HSS) score, as well as inflammatory markers including CRP and IL-6, were adopted to compare and analyse the recovery of knee function in the two groups of patients following surgery.

**Results:**

All participants were followed up for a period exceeding three months. No cases of vascular or nerve injuries were observed during surgery in either group. Moreover, there was no statistically significant difference in total blood loss volume throughout treatment or haemoglobin or haematocrit levels (*P* > 0.05). furthermore, Group A underwent a shorter operation than Group B did (*P* < 0.05). Group B demonstrated decreased postoperative visual analog scale (VAS) pain levels, calf swelling (*P* < 0.05), increased early knee range of motion (*P* < 0.05), and diminished release of blood inflammation markers(IL-6 and CRP) (*P* < 0.05).

**Conclusion:**

The application of tourniquets in HTO surgery reduces intraoperative blood loss and shortens the operative time yet does not substantially affect total bleeding. Nonetheless, the absence of a tourniquet resulted in reduced postoperative pain and facilitated early rehabilitation of knee function.

## Introduction

Knee osteoarthritis (KOA) is an orthopedic disease of the knee that can result in pain and limited movement due to joint degeneration, long-term overload or internal and external rotation of the knee and can significantly impact the patient’s quality of life [1]. During the weight-bearing phase, the knee joint experiences a brief medial retraction, causing most of the load to concentrate on the medial side. Consequently, when there is an accompanying varus deformity of the knee, the load on the medial compartment further increases [2, 3], which results in a series of issues, including cartilage wear on the joint surface, inflammation, and bone redundancy, which in turn engender KOA.

The main treatment for KOA is joint preserving approaches should be utilized as first line treatment, which is based on the severity of the patient’s condition, the risk of treatment, the patient’s own needs, and other factors [4]. However, when patients experience severe symptoms, such as persistent knee pain and functional limitations that affect their daily lives and activities, and when conservative treatments (such as physical therapy and medication) have not provided relief, or when there are significant joint deformities leading to uneven weight bearing on the knee, surgical intervention is warranted [5]. High tibial osteotomy (HTO) is an effective surgical technique employed for the treatment of knee osteoarthritis associated with varus deformity [6]. The clinical use of HTO surgery for the correction of lower extremity force lines has been illustrated, and the stress load concentrated in the medial compartment has been adjusted to the lateral side [7].Despite the fact that HTO is an effective treatment method that can delay or avoid total knee replacement by realigning the knee’s load-bearing axis, the procedure is associated with complications including intraoperative bleeding, postoperative pain, and swelling [8]. Consequently, investigating strategies to minimize these adverse effects during surgery, especially in deciding whether to utilize a tourniquet, may contribute to better postoperative recovery and improve patients’ quality of life [9–12].

Tourniquets have been widely adopted in orthopedic surgery for a long period from the past to the present. In orthopedic surgery, the utilisation of tourniquets allows for bloodless procedures (It refers to the techniques and methods used during medical or surgical procedures to minimize or avoid bleeding.), enhances the visualization of crucial anatomical features, as well as reduces both the anesthesia and operation duration. Historically, joint surgeons routinely applied a tourniquet during total knee arthroplasty (TKA) [13]. The belief was that the application of a tourniquet decreased both intra-operative blood loss and operation duration. Nevertheless, the extensive utilisation of a tourniquet during TKA has generated controversy and can result in certain problems, including edema, discomfort, and delayed wound healing [14–17]. As other measures of preventing blood loss are being widely adopted, there is increasing evidence that not employing tourniquets yields better results [18]. Moreover, research has demonstrated that the application of a tourniquet during arthroscopic anterior cruciate ligament reconstruction did not reduce the extent of intra-articular hemorrhage or the intensity of pain [19]. The use of a tourniquet resulted in a substantial increase in intra-articular bleeding in the early postoperative period [20]. As described above, the utilization of tourniquets results in traditional issues [21] including skin and nerve damage, rhabdomyolysis, deep vein thrombosis, or compartment syndrome, as well as generates dispute in its role of reducing hemorrhage. These conflicting results necessitate further investigation into the effectiveness of tourniquet use in particular surgical procedures, including HTO, particularly regarding its impact on knee function recovery and postoperative complications.

In contemporary medicine, minimizing surgical complications and enhancing surgical safety are critical goals [22]. Since the use of a tourniquet is associated with several potential complications, including skin and nerve injury and deep vein thrombosis [23], studying its necessity in HTO could contribute to the development of safer surgical protocols and the reduction of postoperative complications. Specifically, avoiding unnecessary interventions during surgery, including the use of a tourniquet, may facilitate faster functional recovery. For knee surgery, achieving early functional recovery is a primary concern for patients. If the use of a tourniquet leads to increased postoperative pain and swelling, it could hinder early mobility and delay functional recovery, potentially resulting in prolonged hospital stays, diminished quality of life, and even the occurrence of further complications [24]. Comprehending the specific impact of tourniquet use on postoperative recovery in HTO could assist in medical teams providing personalized treatment plans to maximize recovery outcomes. Despite the fact that research in TKA and other surgeries has questioned the efficacy of tourniquet use, whether these conclusions apply to HTO remains unclear. Consequently, specialized studies on HTO are crucial for validating these findings. Drawing on research from other types of knee surgeries, further validation of tourniquet use in HTO will provide clinicians with more dependable guidance for preoperative decision-making.

This study aimed to assess the impact of tourniquet use during HTO on blood loss, complication rates, and early knee function recovery. Based on the effects observed in TKA procedures, it was hypothesized that the use of a tourniquet in HTO would be unnecessary. Specifically, it was anticipated that tourniquet application would not reduce total blood loss during treatment and could potentially exacerbate postoperative swelling and pain, thereby delaying the early restoration of functional mobility.

## Materials and methods

### Patients

This study, which was designed to be prospective and randomized, received approval from the local institutional review board (YX2022-010). Before the operation, all the subjects provided informed consent. Participants who received HTO treatment at the same hospital between February 2019 and June 2021 were assessed according to defined inclusion and exclusion criteria.

The inclusion criteria.

(1) Patients who were less than 65 years old (female less than 60 years old);

(2) Patients with osteoarthritis in the medial compartment of the knee and a Kellgren–Lawrence classification ≥ grade 2;

(3) Medial proximal tibial angle less than 85°;

(4) Unilateral HTO.

The exclusion criteria:

(1) Patients with severe comorbid conditions, including a prior history of malignancy, diabetes mellitus, renal insufficiency, or peripheral neurovascular disease.

(2) Patients with a prior history of thromboembolism or a predisposition to thromboembolism.

(3) Preoperative hemohaemoglobin less than 90 g/L.

(4) Patients with preoperative coagulation disorders or previous aspirin use.

The 86 patients mentioned above were Stratified Randomization (Patients were stratified based on factors including age and gender, and then randomly assigned to groups within each stratum.) divided into two groups, A and B, with 43 patients in each group. Group A consisted of patients who underwent HTO with a tourniquet, while group B included those who underwent the procedure without a tourniquet. The research staff responsible for the assessments were blinded to the group assignments. Meanwhile, the group assignment was concealed from the research workers conducting the assessments. Besides, the patients in both groups underwent preoperative frontal and lateral radiographs of the affected knee and full-length radiographs of both lower limbs. Three months after surgery, there was no attrition in either group.

### Operation

Both groups of patients underwent surgery under general anesthesia. All procedures were performed by the same surgeon and involved medial open wedge high tibial osteotomy.

After general anesthesia, an inflatable tourniquet was placed at the root of the thigh. In group A, the tourniquet was inflated to greater than 100 mmHg systolic pressure before the skin incision was made based on the Association of Operating Room Nurses recommendations [25], and in group B, the tourniquet was not inflated. An 8-cm surgical incision was made on the pes anserinus of the affected limb, extending toward the posterior medial corner of the tibial plateau. The proximal tibia was then dissected layer by layer to expose the tibial surface, with the posterior edge of the tibia being revealed by releasing the superficial layer of the medial collateral ligament. Following the accurate positioning via C-arm fluoroscopy, the osteotomy was completed according to the standard HTO procedure. Meanwhile, Titanium plate screws (Johnson & Johnson, USA) were adopted to fix the osteotomy site upon the intended angle was opened. In both groups, tranexamic acid (TXA) was applied to stop bleeding. After the incision was closed, a solution containing 2 g of TXA was prepared by dissolving the drug in 30 milliliters of saline. Upon surgery, a large amount of saline was flushed, the TXA solution was subsequently injected into the osteotomy site, the sutures were placed layer by layer, the drains were left in place, and the incision was sutured, and an elastic bandage was applied with pressure to the area.

### Postoperative treatment

Patients in both groups began functional knee flexion and extension exercises, as well as quadriceps strengthening exercises, on the second postoperative day under the supervision of medical staff. Supported abduction to the floor was also encouraged. The criteria for patient discharge were as follows:

(1) Perform a straight leg raise and hold the position for a duration of 30 s.

(2) A maximum knee flexion of 90° is achievable.

(3) There were no signs of anemia, fever, nausea, impaired wound healing, or any other related complications.

### Outcome measures

Intraoperative complications, drainage volume, operative time and length of hospital stay were recorded for both groups. Pre- and postoperative complete blood counts were measured, and haematocrit (Hct) and haemoglobin (Hb) indices were recorded. Besides, Serum concentrations of C-reactive protein(CRP) and IL-6 were assessed both before and three days after surgery and were subsequently duly documented. The combination of CRP and IL-6 offers a more thorough evaluation of the inflammatory state. CRP, an acute-phase protein synthesized by the liver, reflects the overall level of systemic inflammation [26], while IL-6, a pleiotropic cytokine, provides insights into more specific inflammatory processes and immune regulation [27]. As a result, both are frequently chosen as key indicators in studies related to inflammation. The gross equation was employed to calculate the patient’s predicted blood volume (PBV) [28], and the total estimated blood loss was calculated based on the formula described in prior studies [29]. The formula is described as follows:

$$\:PBV\hspace{0.17em}=\hspace{0.17em}K1\times\:Height\:\left(m\right)^{3}+K2\times\:Body\:weight\:\left(kg\right)\hspace{0.17em}+\hspace{0.17em}K3$$

$$\:Males:\:K1\hspace{0.17em}=\hspace{0.17em}0.3669,\:K2\hspace{0.17em}=\hspace{0.17em}0.03219,\:K3\hspace{0.17em}=\hspace{0.17em}0.6041$$

$$\:Female:\:K1\hspace{0.17em}=\hspace{0.17em}0.3561,\:K2\hspace{0.17em}=\hspace{0.17em}0.03308,\:K3\hspace{0.17em}=\hspace{0.17em}0.1833$$

$$\:Total\:blood\:loss\:volume\hspace{0.17em}=\hspace{0.17em}PBV\times\:(pre-Hct\:-\:post-Hct)$$

$$\eqalign{& {\rm{Hidden}}\>{\rm{blood}}\>{\rm{loss}}\>\left( {{\rm{HBL}}} \right){\rm{ = }} & \cr & {\rm{Total}}\>{\rm{blood}}\>{\rm{loss}}\>{\rm{ - }} \cr & {\rm{(Intraoperative}}\>{\rm{blood}}\>{\rm{loss + Drainage}}\>{\rm{volume)}} \cr}$$

Note intraoperative haemorrhage comprises two components: (1) the volume of blood extracted by suction (the volume of fluid in the suction minus the volume of irrigation fluid), (2) the volume of blood removed by gauze during the operation (calculated based on the distinctions in the weight of the gauze prior to and following the operation), and the sum of these two components represents the total intraoperative hemorrhage. The drainage volume refers to the volume of bloody fluid that is drained from the drainage tube after surgery.

Knee range of motion (ROM) and pain level were assessed using a visual analog scale (VAS) following exercise, and maximal calf circumference was documented on the first, third, fifth, and thirtieth days following surgery and postoperative Hospital for Special Surgery (HSS) score. The evaluations were conducted and assessed by a single investigator who had received training and was blinded to the distribution of the assessments. Similarly, the data analyst was unaware of the group assignments.

### Statistical analysis

SPSS 18.0 (SPSS, Chicago, IL, USA) statistical software was adopted for statistical analysis of the collected data. The measurement data are shown as the mean ± the standard deviation, whereas the count data are represented by the number of patients. Except for that, the measurement data were tested for variance homogeneity using the Levene’s test. Differences between groups were analyzed using independent sample t-tests. Additionally, chi-square tests were employed to evaluate the differences among the groups. A significance level of *P* < 0.05 was used to determine statistical significance.

## Results

A total of 86 patients (43 per group) were included in the analysis, with no significant differences in baseline characteristics such as age, BMI, or preoperative scores (*P* > 0.05). Please refer to Table 1 for detailed preoperative data.

**Table 1:** Preoperative characteristics of patients

	Group A (*n* = 43)	Group B (*n* = 43)	*P*
Age (years)	56.28 ± 6.48	57.53 ± 5.85	0.392
Gender (male/female)	11/32	14/29	0.476
BMI (kg/m^2^)	24.32 ± 1.62	24.15 ± 1.36	0.597
Preop VAS score	4.77 ± 0.61	4.86 ± 0.64	0.492
Preop HSS score	58.58 ± 8.28	58.26 ± 8.22	0.855
Preop Hb (g/L)	129.12 ± 11.87	132.56 ± 12.54	0.195
Preop Hct (%)	39.09 ± 3.59	39.67 ± 3.81	0.469
Preop CRP (mg/L)	7.622 ± 1.23	7.81 ± 0.74	0.451
Preop IL-6 (pg/mL)	5.122 ± 2.23	5.31 ± 1.24	0.672

BMI: body mass index; Preop: preoperative; VAS: visual analog scale; HSS: Hospital for Special Surgery; Hb: hemohaemoglobin, Hct: haematocrit; CRP: C-reactive protein

**Table 2:** Operative data of patients

	Group A(*n* = 43)	Group B(*n* = 43)	*P*
Operation time (min)	100.86 ± 32.84	115.72 ± 20.33	**0.014**
Total blood loss (ml)	359.22 ± 81.99	379.77 ± 86.96	0.263
Intraoperative blood (ml)	130.23 ± 43.83	176.74 ± 64.87	**< 0.001**
Apparent bleeding (ml)	233.02 ± 55.61	250.58 ± 67.47	0.192
Hidden blood loss (ml)	126.20 ± 71.96	129.19 ± 80.70	0.857
Volume of drainage (ml)	102.79 ± 51.75	77.09 ± 33.72	**0.008**
Postop Hb (g/L)	104.93 ± 10.83	102.37 ± 10.40	0.195
Postop Hct (%)	30.14 ± 3.07	30.30 ± 3.51	0.819
Postop CRP (mg/L)	83.86 ± 4.67	55.14 ± 5.47	**0.003**
Postop IL-6 (pg/mL)	131.14 ± 17.37	92.14 ± 12.14	**< 0.001**

Postop: postoperative, Hb: haemoglobin, Hct: haematocrit. CRP: C-reactive protein.The bold font indicates statistically significant

Neither group experienced any transfusion events or intraoperative vascular or nerve injuries. For surgical variables, the operation time of group A was 100.86 ± 32.84 min, and that of group B was 115.72 ± 20.33 min. Moreover, the operation time with a tourniquet was substantially shorter than that without a tourniquet (*P* < 0.05). The A group had significantly less intraoperative blood than did the B group (130.23 ± 43.83 ml vs. 176.74 ± 64.87 ml, *P* < 0.05). Nonetheless, the drainage volume was significantly higher in group A compared to group B (102.79 ± 51.75 ml vs. 77.09 ± 33.72 ml, *P* < 0.05). Furthermore, the inflammatory marker, CRP and IL-6 levels in group A were markedly elevated compared to those in group B. The study results showed that group A had the highest levels of CRP and IL-6, as depicted in Fig. 4. However, no significant differences were observed in total blood loss, apparent bleeding, hidden blood loss, postoperative Hb, or postoperative Hct (*P* > 0.05). Detailed information can be found in Table 2.

**Fig. 1:**
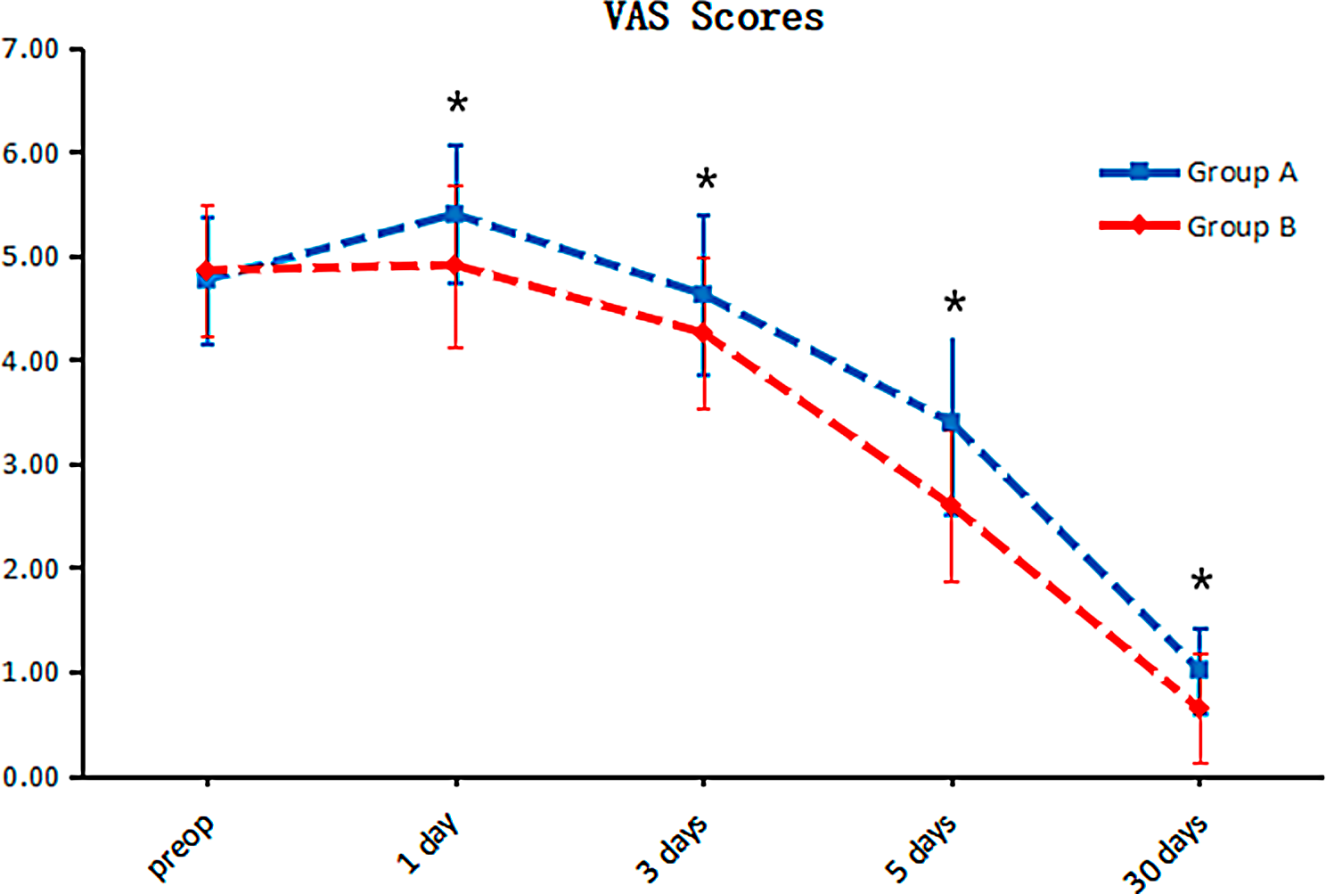
Levels of postoperative serum inflammatory factors. The levels of serum CRP (*P* = 0.03) and IL-6 (*P* < 0.001) were significantly greater in group A than in group B after surgery. Group A consisted of patients who received tourniquets, while Group B consisted of patients who did not receive tourniquets

**Fig. 2:**
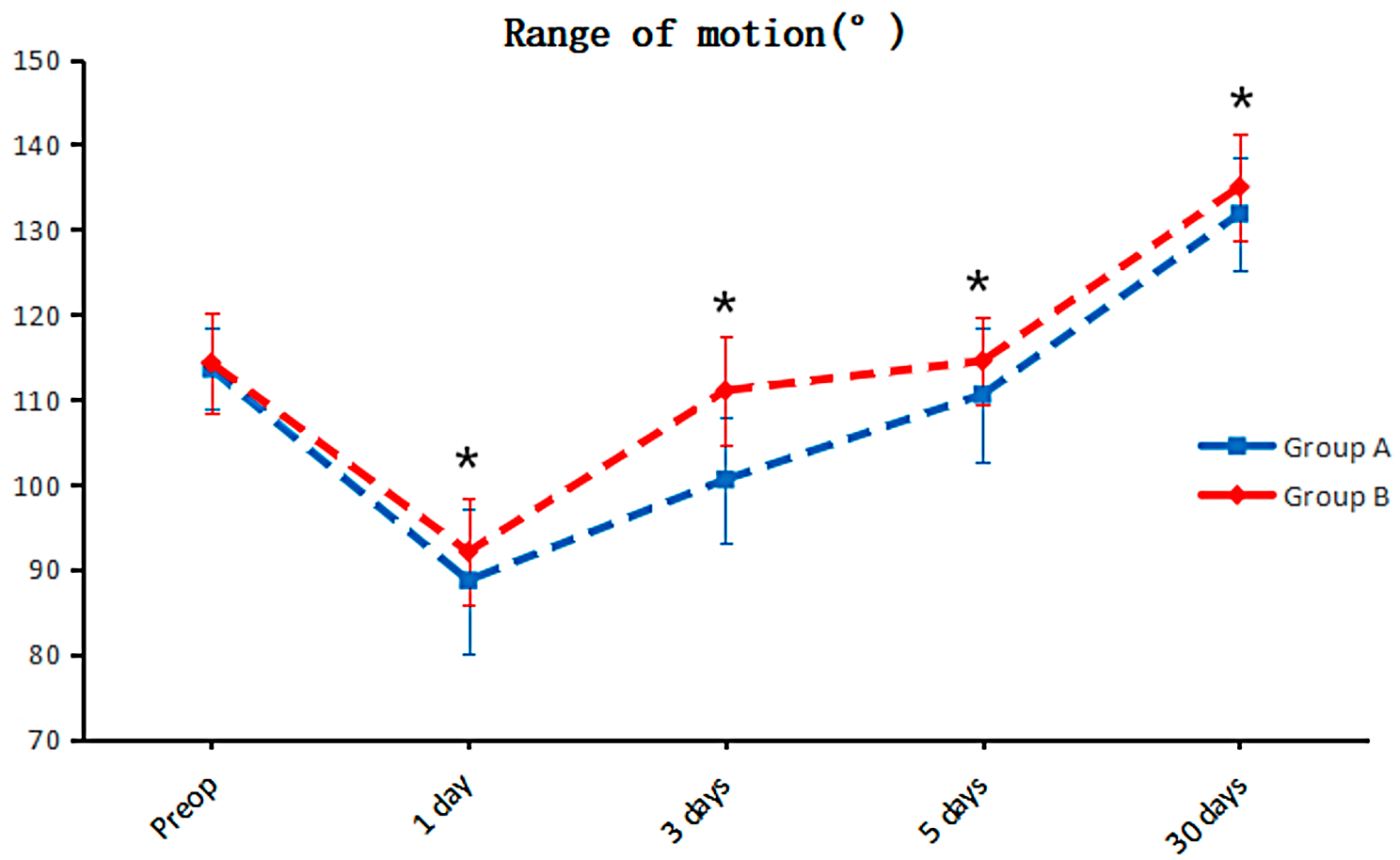
Fluctuations in VAS scores throughout the perioperative period. The VAS scores of group B were significantly lower than those of group A on the first day (*P* = 0.002), third day (*P* = 0.022), fifth day (*P* < 0.001) and one month (*P* < 0.001) after surgery. Group A consisted of patients who received tourniquets, while Group B consisted of patients who did not receive tourniquets

**Fig. 3:**
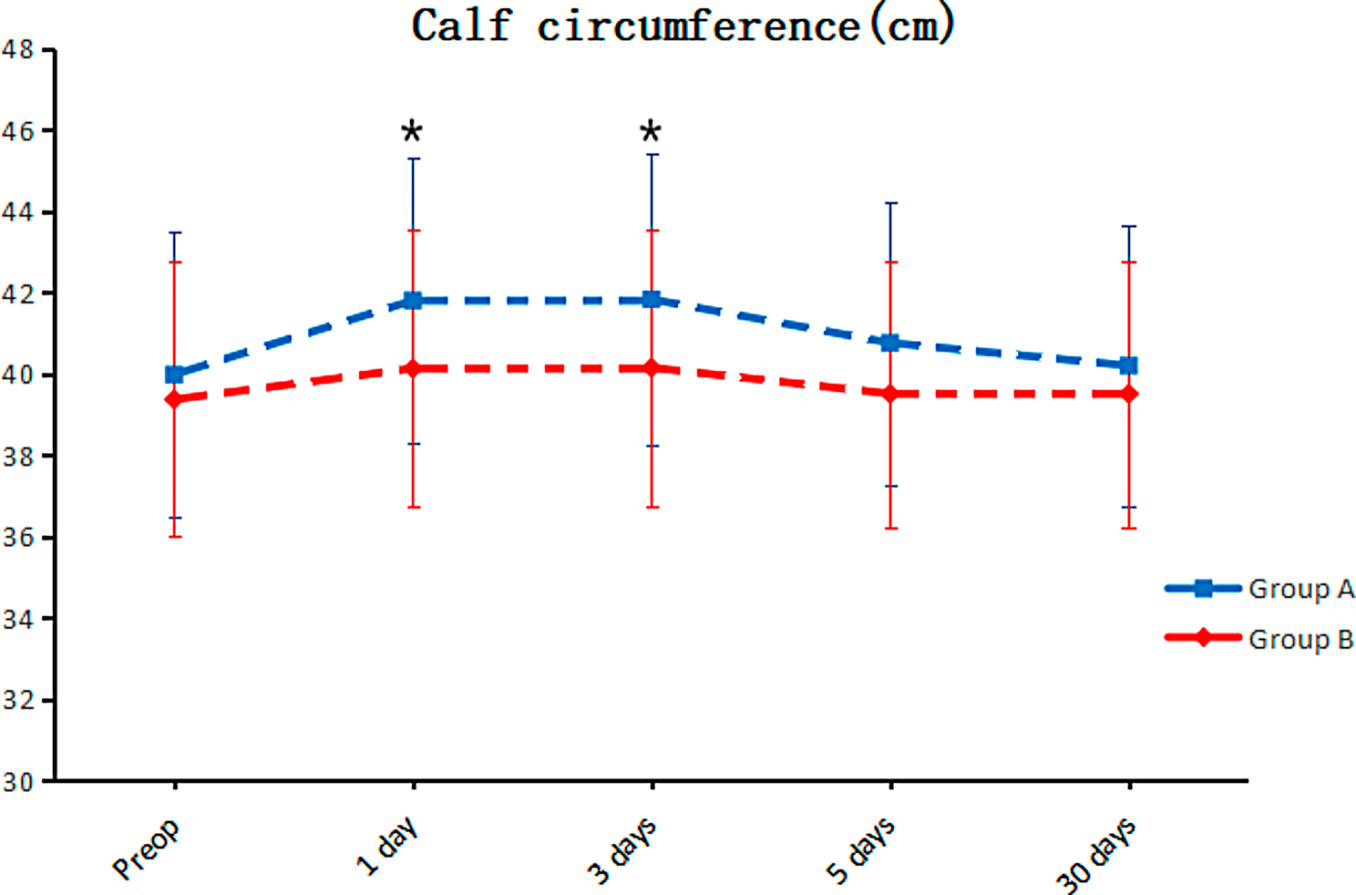
Alterations in the extent of movement throughout the perioperative phase. The range of motion exhibited by Group B was significantly greater than that exhibited by Group A on the first day (*P* = 0.040), third day (*P* < 0.001), fifth day (*P* = 0.007) and one month (*P* = 0.027) after surgery. Group A consisted of patients who received tourniquets, while Group B consisted of patients who did not receive tourniquets

Group B exhibited substantially lower VAS scores on the days following the surgical procedure (one, three, five, and thirty) (*P* < 0.05), as demonstrated in Fig. 1. Notably, knee mobility increased during the days following surgery (one, three, five, and thirty days) (*P* < 0.05). Group B also indicated a reduction in calf circumference on postoperative days one and three (*P* < 0.05), as illustrated in Fig. 2. No statistically significant difference in calf circumference was detected between the groups on postoperative days five and thirty (*P* > 0.05), as revealed in Fig. 3. Furthermore, no significant differences were observed in postoperative HSS scores at thirty days or three months (*P* > 0.05). The details are provided in Table 3.

**Fig. 4:**
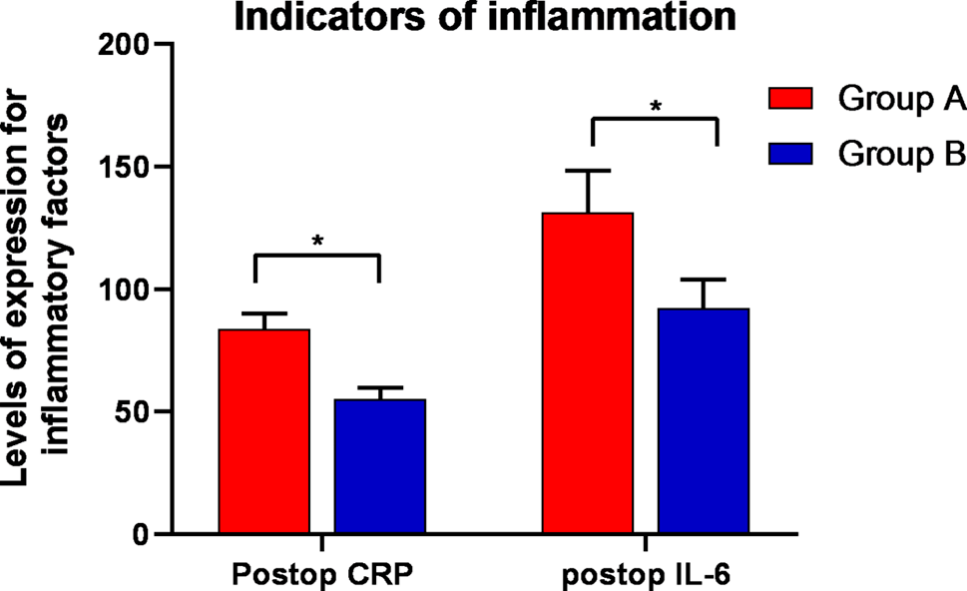
Fluctuations in the diameter of the calf throughout the surgical procedure. The calf circumference of group B was significantly lower than that of group A on both the first (*P* = 0.028) and third days (*P* = 0.029) after surgery. Group A consisted of patients who received tourniquets, while Group B consisted of patients who did not receive tourniquets

**Table 3:** Postoperative outcomes of patients

	Group A(*n* = 43)	Group B(*n* = 43)	*P*
Postop VAS score			
1 day	5.40 ± 0.66	4.91 ± 0.78	**0.002**
3 days	4.63 ± 0.76	4.26 ± 0.73	**0.022**
5 days	3.40 ± 0.88	2.60 ± 0.73	**<0.001**
30 days	1.02 ± 0.41	0.65 ± 0.53	**<0.001**
Postop ROM (°)			
1 day	88.72°±8.53°	92.09°±6.29°	**0.040**
3 days	100.58°±7.42°	111.05°±6.32°	**<0.001**
5 days	110.58°±7.88°	114.53°±5.10°	**0.007**
30 days	131.86°±6.73°	135.00°±6.17°	**0.027**
Postop calf girth (cm)			
1 day	41.80 ± 3.52	40.13 ± 3.40	**0.028**
3 days	41.83 ± 3.57	40.15 ± 3.40	**0.029**
5 days	40.76 ± 3.48	39.51 ± 3.28	0.092
30 days	40.20 ± 3.44	39.51 ± 3.28	0.346
Postop HSS score			
30 days	84.60 ± 2.74	85.67 ± 2.25	0.051
3 months	88.23 ± 2.65	87.56 ± 2.43	0.223

Postop: postoperative, VAS: visual analog scale, ROM: range of motion, HSS: Hospital for Special Surgery. Bold font signifies statistically significant

## Discussion

The key finding of this study was that the application of a tourniquet during HTO did not reduce postoperative blood loss. On the contrary, the use of a tourniquet was associated with increased postoperative pain, delayed functional recovery of the knee joint, and elevated levels of inflammatory mediators. A number of recent studies have shown that not utilizing a tourniquet in TKA surgery is superior to employing a tourniquet [14, 16, 30–32]. Nonetheless, there is no concrete conclusion as to whether this is also true in HTO surgery.

As previously mentioned, HTO surgery primarily involves open osteotomy, a highly invasive procedure that typically results in significant intraoperative bleeding. Moreover, due to the fact that KOA patients are mostly elderly people, they usually have more underlying diseases, especially cardiovascular and cerebrovascular diseases, accordingly, it is challenging to tolerate postoperative anemia attributable to bleeding. Hence, intraoperative hemostasis in KOA patients is a very urgent issue.

At the outset, the application of tourniquets resulted in a notable decrease in intraoperative blood loss during HTO surgery, which improved intraoperative visibility, reduced surgical complexity, and shortened the duration of the procedure. Nonetheless, tourniquets also increase the risk of thrombosis and a number of other complications.

The effectiveness of tourniquets in reducing blood loss during surgery remains a subject of ongoing debate among researchers. Numerous studies have indicated that intraoperative tourniquet use delays postoperative functional recovery, increases pain, and increases postoperative blood loss (Volume of drainage). Nevertheless, some studies have also demonstrated that the utilisation of tourniquets during surgery significantly reduces blood loss and does not adversely affect early postoperative outcomes. A study conducted by Goel R et al. involving 200 patients who underwent TKA demonstrated that the use of a tourniquet significantly reduced blood loss during the procedure and did not adversely affect the initial postoperative outcomes [33]. The application of a tourniquet in standard TKA procedures is both safe and effective, and concerns regarding its potential adverse effects on functionality and pain may be unwarranted.

Apparently, in this study, patients who adopted tourniquets had more severe postoperative knee pain and markedly greater postoperative VAS scores than did those who did not use tourniquets, as indicated in Fig. 1. Furthermore, patients who underwent HTO with a tourniquet exhibited reduced knee mobility compared to those who did not use a tourniquet, along with increased postoperative swelling in the lower extremities, as demonstrated in Figs. 2 and 3. According to Fig. 4, the inflammatory reaction was more severe in patients who utilized a tourniquet during surgery. Elevated intraoperative inflammatory markers frequently indicate the strong response of the body to surgical trauma [34]. If the inflammatory factor is markedly elevated or persists at elevated levels postoperatively, it may indicate the potential for surgical complications, namely, infection or tissue damage. This factor is pivotal in the assessment of the patient’s recovery and prognosis [35].These differences may be caused by ischemia in the operated limb due to tourniquet use and triggered ischemia–reperfusion injury following surgery completion. The difference in postoperative HSS scores between the two groups was not statistically significant, likely due to the more complex nature of the HSS scoring system, which may have introduced bias during follow-up and influenced the results.

The use of a tourniquet during surgery may damage cells through the following mechanisms: On the condition that a tourniquet is inflated, blood flow to the affected limb stops, resulting in a lack of oxygen and nutrients in the area, which attribute to changes in the cells, including a decrease in ATP levels, an increase in acidity within the cells, an excessive amount of calcium inside the cells, and damage to the cells [36]. Several studies have demonstrated that the use of tourniquets can induce ischemia‒reperfusion injury, which in turn leads to a reduction in protein synthesis [37] and increased degradation [38] in skeletal muscle cells, as well as upregulation of the expression of genes that regulate cellular stress pathways.

Unexpectedly, the application of tourniquets leads to an increase in the amount of hidden blood loss in patients following surgery. While tourniquets significantly reduce intraoperative bleeding, resulting in a clearer surgical field and shorter operative times, patients who underwent surgery with tourniquet use exhibited a significant increase in postoperative drainage at the surgical site. The total blood loss was subsequently calculated using the gross equation. Moreover, the overall bleeding volume did not significantly differ between the two groups. Possible factors contributing to increased hidden blood loss include: 1. Leakage throughout microvascular and capillary networks. The release of the tourniquet and the subsequent restoration of blood flow to the surgical site may lead to leakage from the microvessels and capillaries in the surrounding tissues. The use of the tourniquet during the procedure exerts additional pressure on the blood vessels, potentially compromising the structural integrity of the vessel walls, which can result in increased blood loss. This blood predominantly seeps into the interstitial space of the tissue rather than manifesting as postoperative haemorrhage, resulting in imperceptible blood loss [39]; 2.Tourniquet severs blood flow to the distal limb, giving rise to the entrapment of blood in non-surgical regions, including the heart and upper limbs. Upon the release of the tourniquet, blood rapidly returns to the surgical site, and part of it may permeate the tissue spaces, resulting in undetectable blood loss [40]; 3. Release of the tourniquet may trigger an inflammatory response in the nearby tissues caused by the surgical trauma, which may facilitate the leakage of plasma components through the microvessel walls [41].Additionally, numerous studies have indicated that the use of tourniquets in TKA surgery is not effective at reducing total blood loss [42–45].

With advancements in perioperative hemostasis techniques, tourniquets are no longer the primary method for controlling bleeding. In addition, tranexamic acid is being increasingly utilized for both intraoperative and postoperative hemostasis, demonstrating greater effectiveness compared to other methods [46]. Gelatin sponges can absorb a large amount of blood and swell themselves to achieve hemostasis via compression. By applying pressure to the tissues in the surgical area using cotton pads and pressure bandages, the exudation and swelling of the operated limb can be mitigated to some extent, thereby promoting the functional recovery of the knee joint post-surgery [47, 48].

This study has several limitations. In the first place, due to personal capacity and time constraints, the sample size of this study was small, with 86 patients. Secondly, this was a prospective controlled study constrained by the use of a pneumatic tourniquet, and the inability to achieve blinding of the study team may have introduced some degree of bias. Third, due to the length of the study, this study lacked long-term follow-up, and there was a lack of data on long-term postoperative functional improvement of the knee joint. Another limitation of this study is the absence of a power analysis to determine the appropriate sample size. As this was a prospective study, conducting a power analysis would have been beneficial in ensuring that the sample size was sufficient to detect meaningful differences between the groups. Future research should incorporate a power analysis to optimize the sample size and enhance the robustness of the findings.

## Conclusion

Notwithstanding these limitations, the findings of this investigation illustrate that employing tourniquets in HTO surgery can reduce merely intraoperative bleeding and thus the operative time yet has no significant effect on the total bleeding volume. Furthermore, the use of tourniquets results in an elevated release of inflammatory mediators, heightened postoperative pain, delayed recovery of knee function, and exacerbated swelling in the lower extremities. Consequently, tourniquets should not be employed as a standard practice in HTO surgery once other hemostasis techniques are applied.

## Data Availability

The datasets used and/or analyzed during the current study are available from the corresponding author upon reasonable request.

## Declarations

### Author declaration

All authors listed meet the authorship criteria according to the latest guidelines of the International Committee of Medical Journal Editors, and that all authors are in agreement with the manuscript.

## Abbreviations

HTO: High tibial osteotomy
VAS: Visual analog scale
KOA: Knee osteoarthritis
TKA: Total knee arthroplasty
TXA: Tranexamic acid
PBV: Predicted blood volume
ROM: Range of motion
BMI: Body mass index
HSS: Hospital for Special Surgery
Hb: Hemoglobin
Hct: Hematocrit
CRP: C-reactive protein
